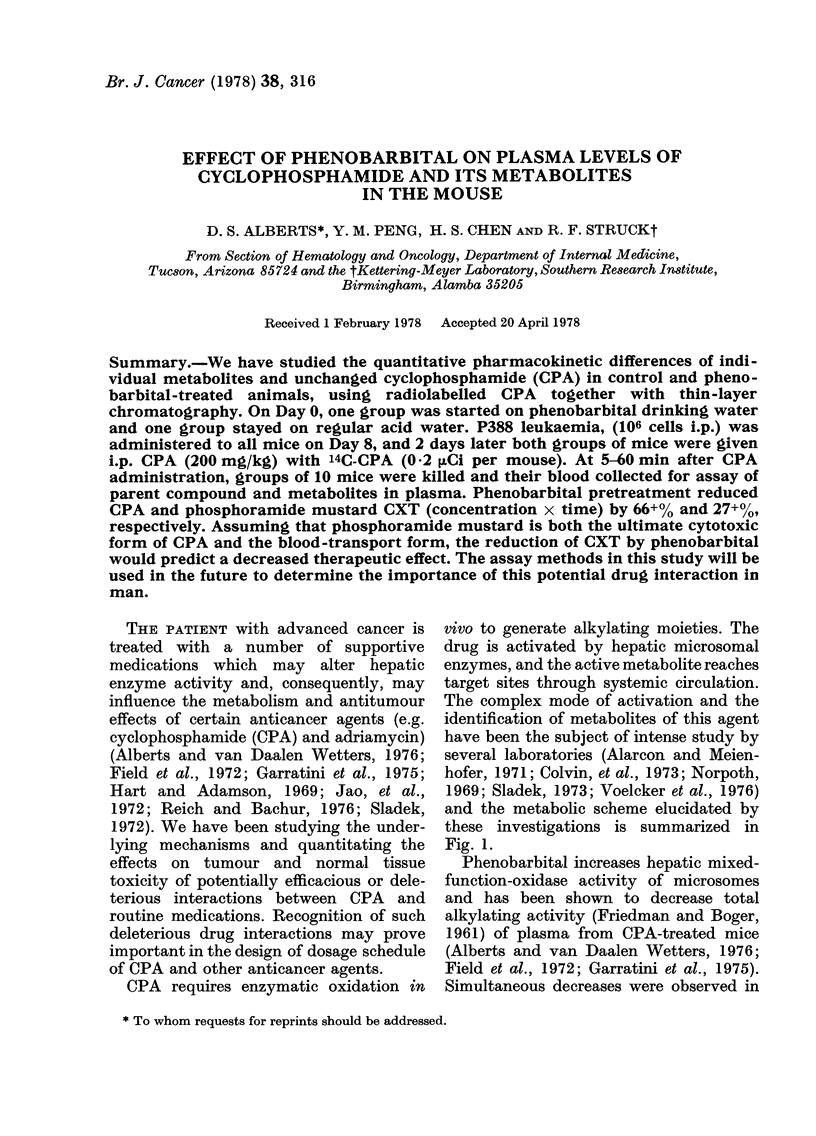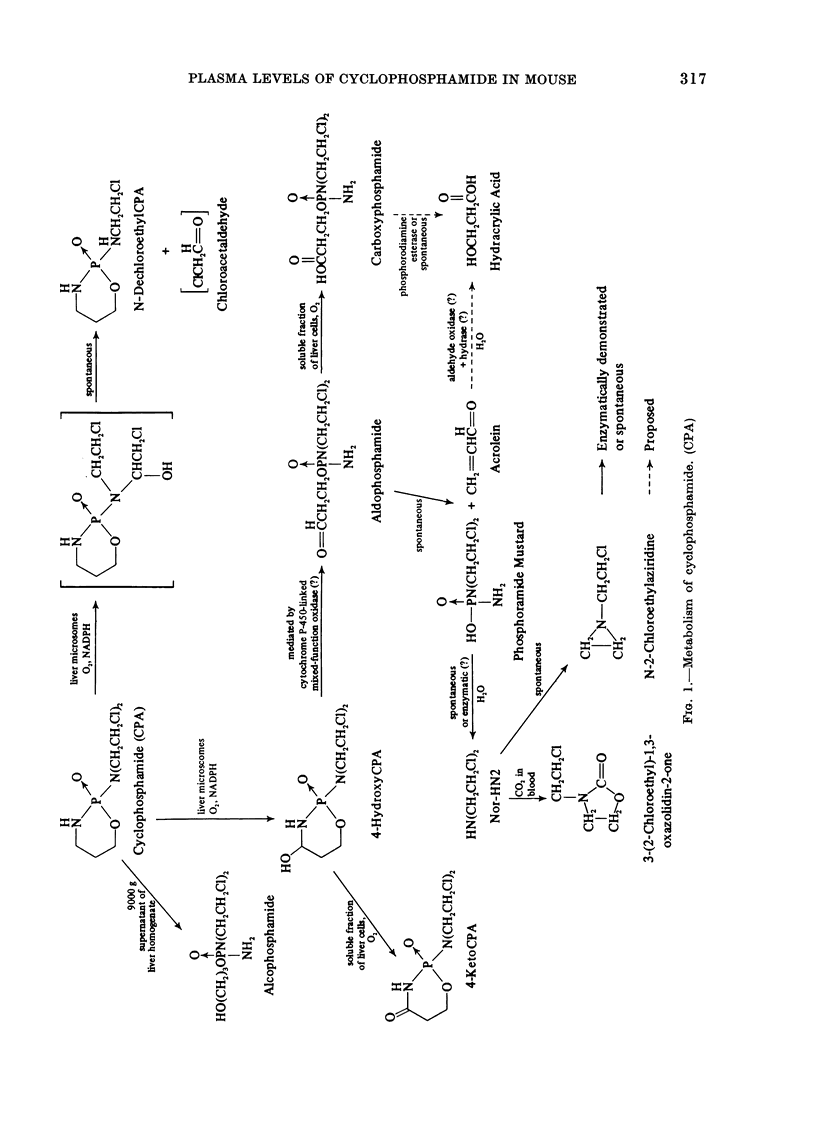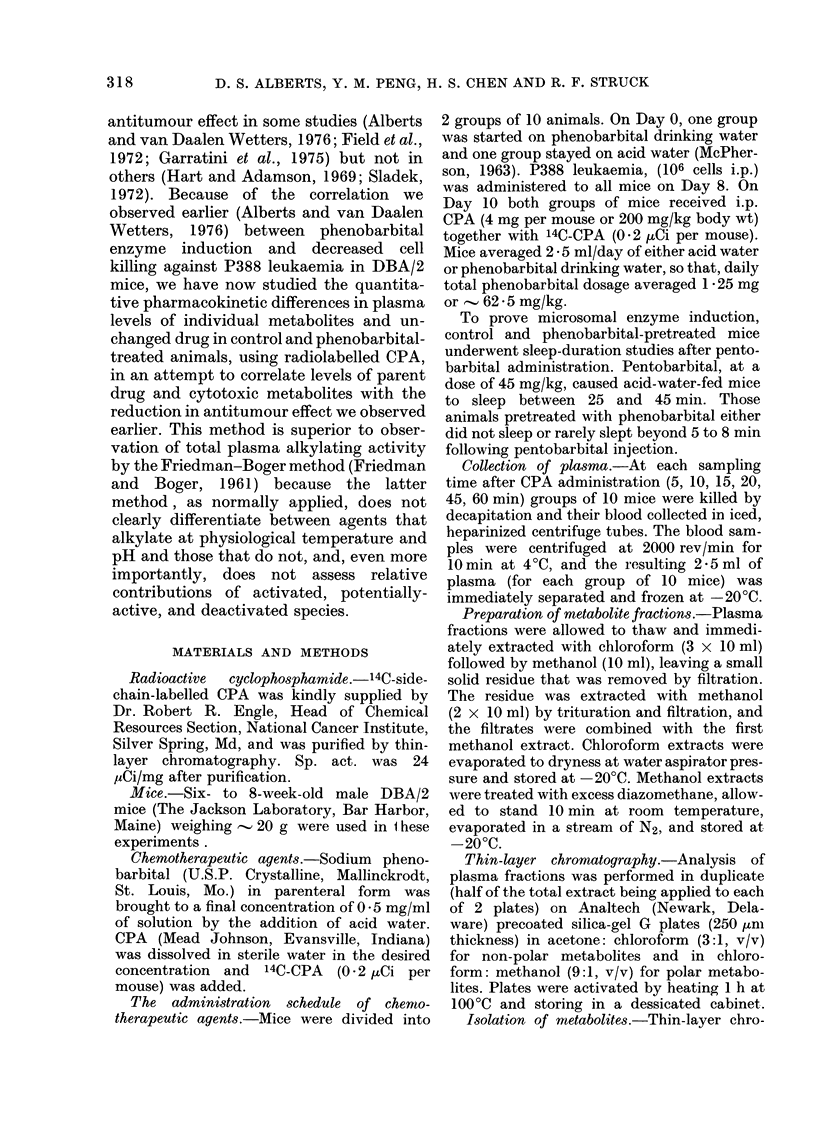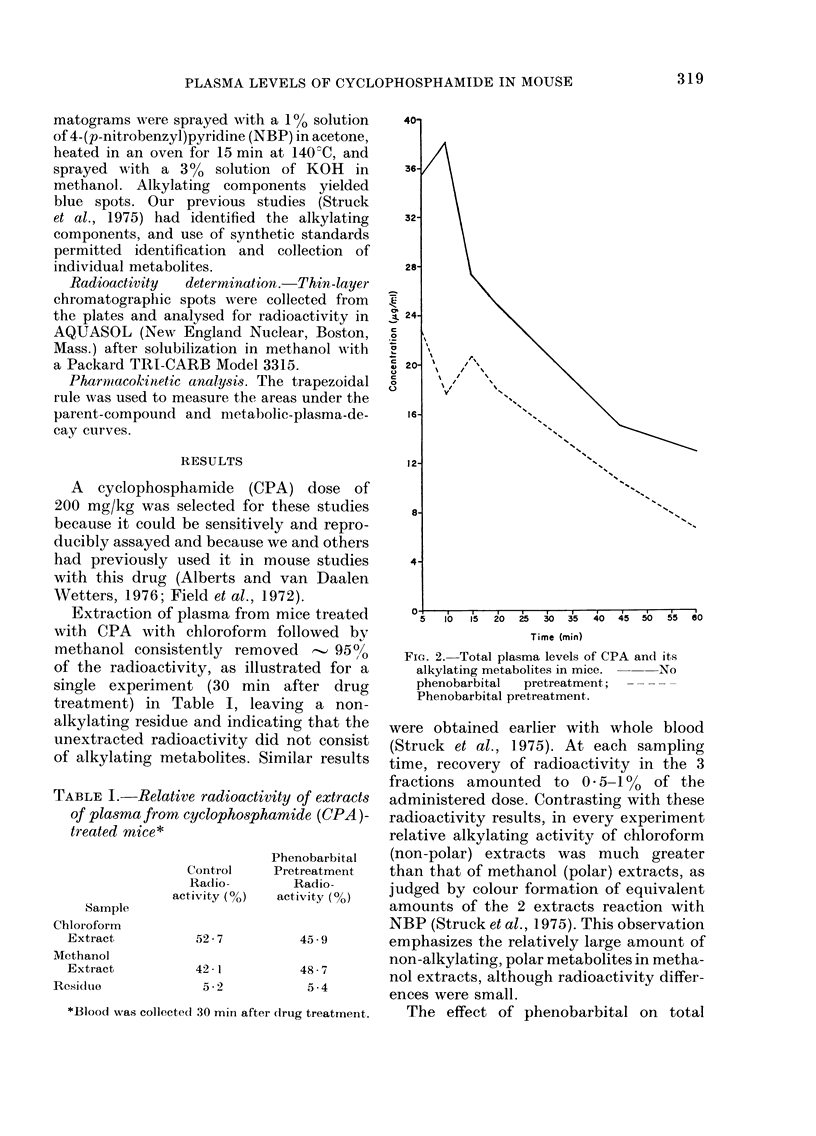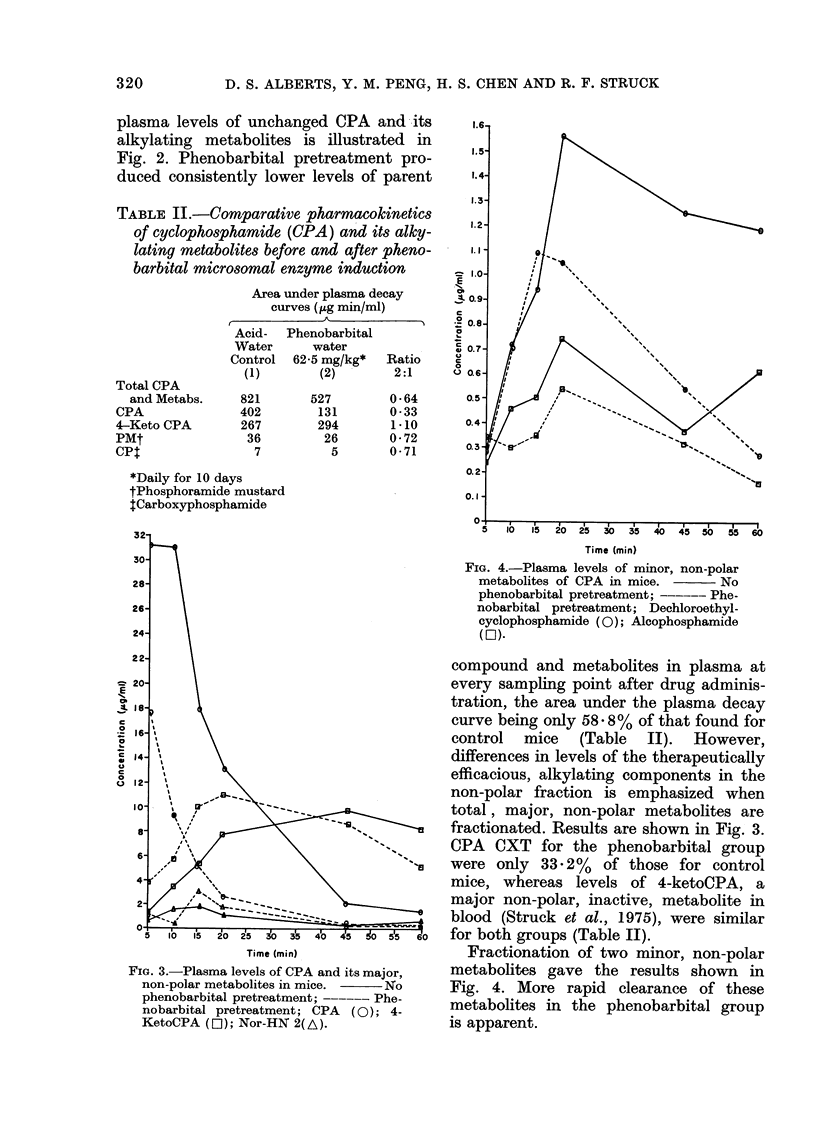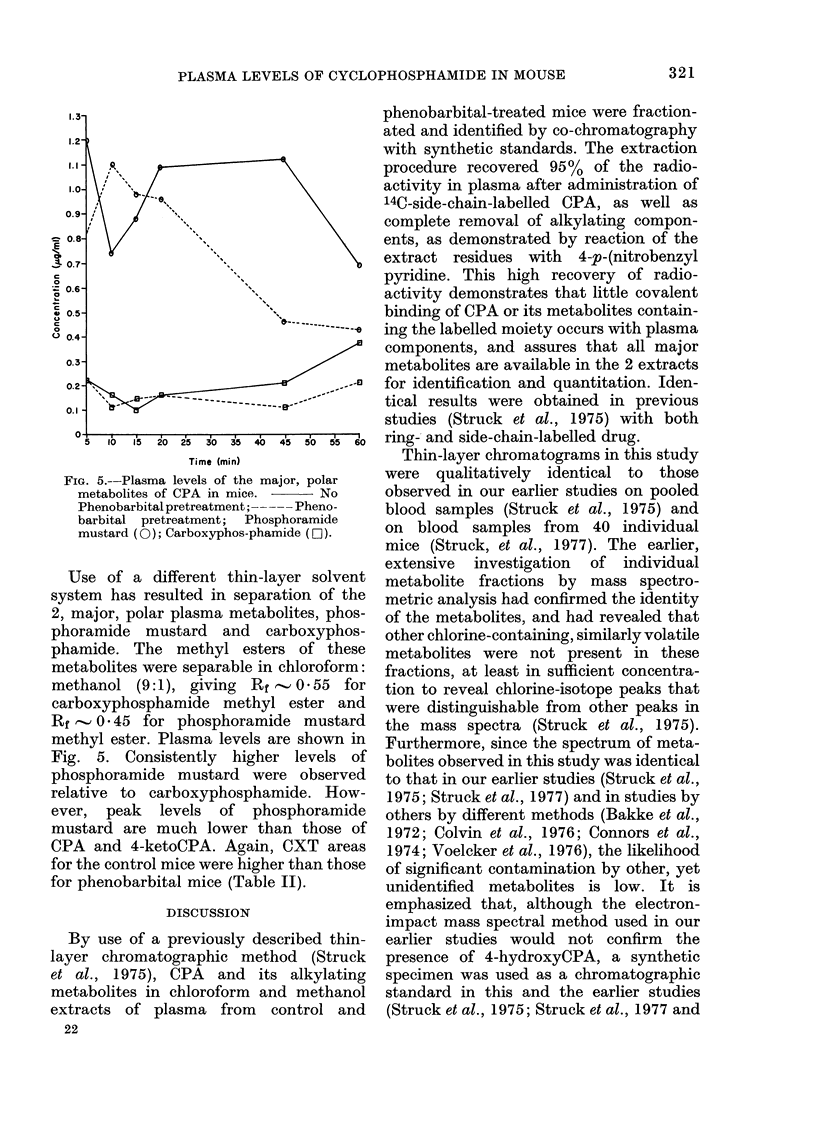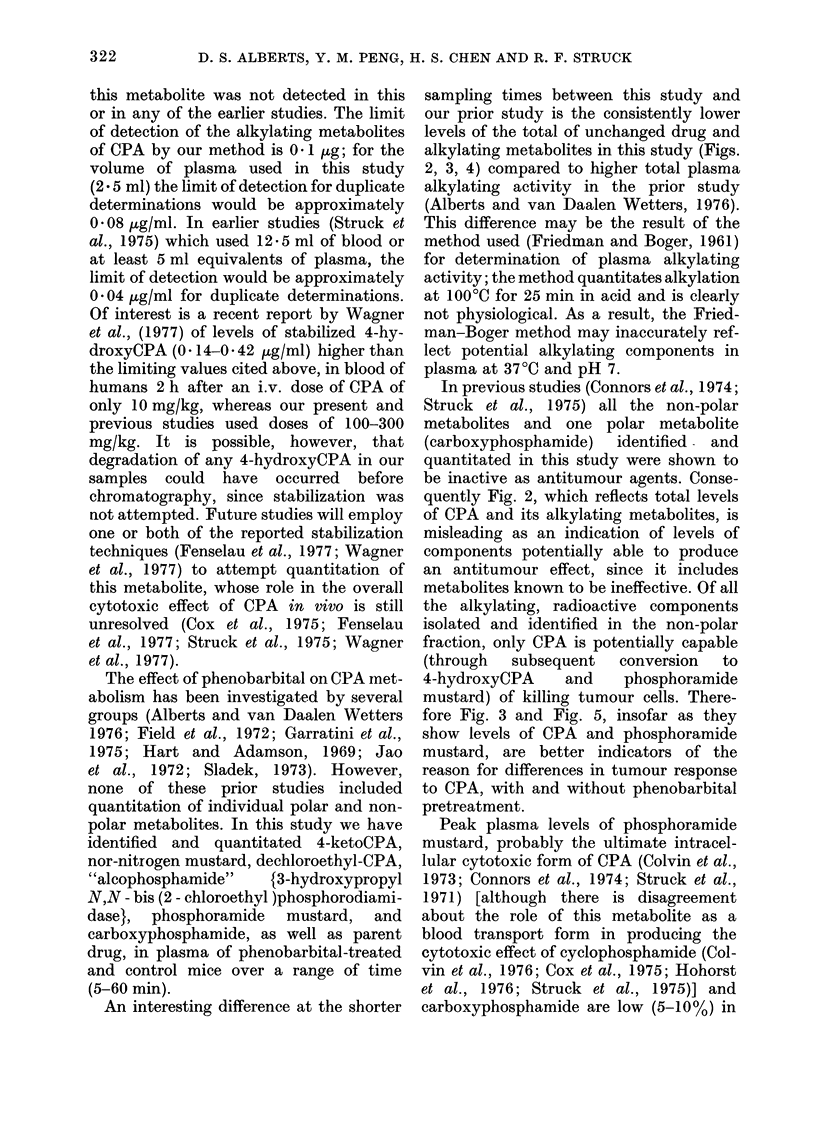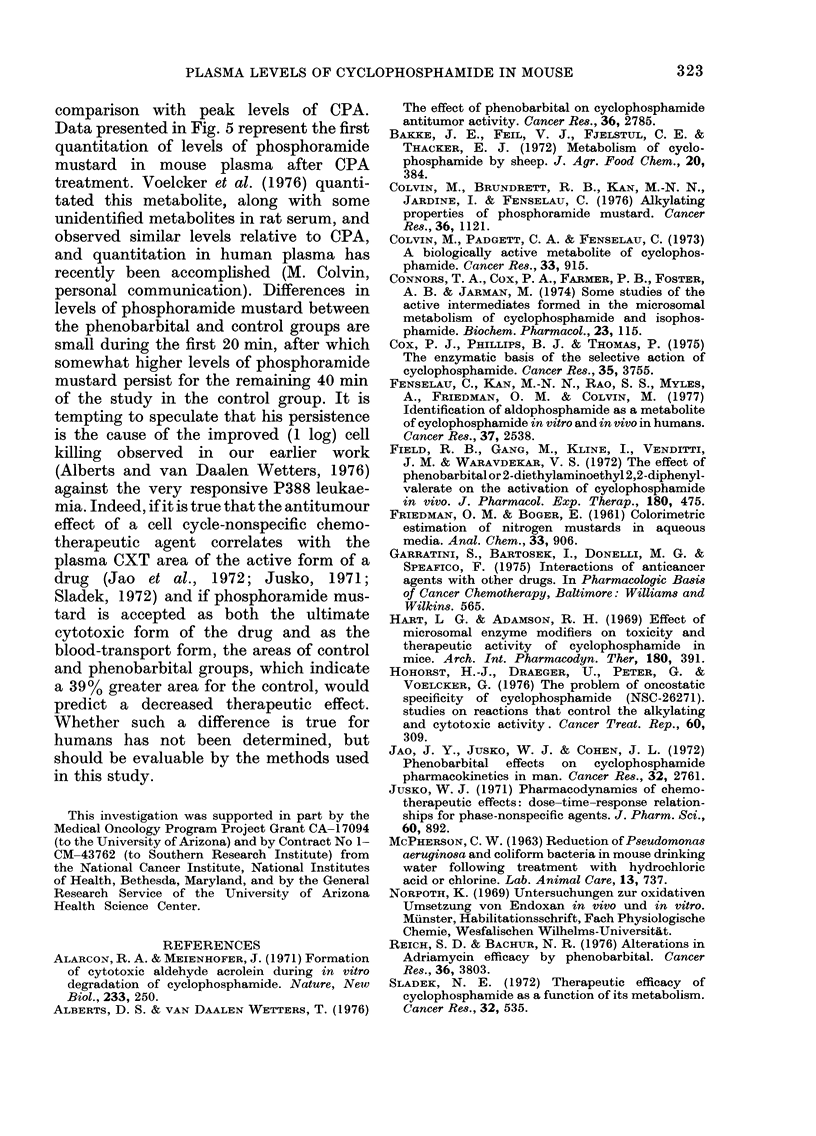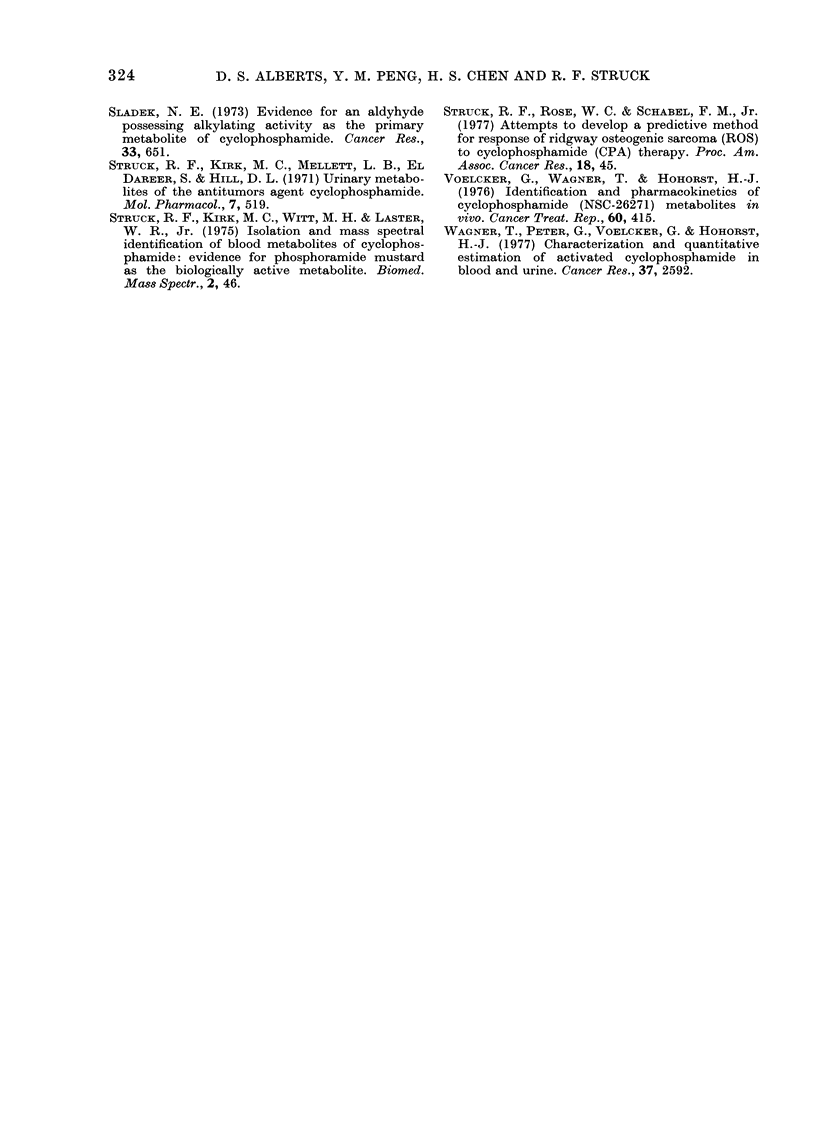# Effect of phenobarbital on plasma levels of cyclophosphamide and its metabolites in the mouse.

**DOI:** 10.1038/bjc.1978.204

**Published:** 1978-08

**Authors:** D. S. Alberts, Y. M. Peng, H. S. Chen, R. F. Struck

## Abstract

We have studied the quantitative pharmacokinetic differences of individual metabolites and unchanged cyclophosphamide (CPA) in control and phenobarbital-treated animals, using radiolabelled CPA together with thin-layer chromatography. On Day 0, one group was started on phenobarbital drinking water and one group stayed on regular acid water. P388 leukaemia, (10(6) cells i.p.) was administered to all mice on Day 8, and 2 days later both groups of mice were given i.p. CPA (200 mg/kg) with 14C-CPA (0.2 muCi per mouse). At 5--60 min after CPA administration, groups of 10 mice were killed and their blood collected for assay of parent compound and metabolites in plasma. Phenobarbital pretreatment reduced CPA and phosphoramide mustard CXT (concentration x time) by 66+% and 27+%, respectively. Assuming that phosphoramide mustard is both the ultimate cytotoxic form of CPA and the blood-transport form, the reduction of CPA by phenobarbital would predict a decreased therapeutic effect. The assay methods in this study will be used in the future to determine the importance of this potential drug interaction in man.


Br. J. Cancer (1978) 38, 316

### EFFECT OF PHENOBARBITAL ON PLASMA LEVELS OF CYCLOPHOSPHAMIDE AND ITS METABOLITES IN THE MOUSE

D. S. ALBERTS*, Y. M. PENG, H. S. CHEN AND R. F. STRUCK†

*From Section of Hematology and Oncology, Department of Internal Medicine, Tucson, Arizona 85724 and the †Kettering-Meyer Laboratory, Southern Research Institute, Birmingham, Alamba 35205*

Received 1 February 1978 Accepted 20 April 1978

**Summary.—We have studied the quantitative pharmacokinetic differences of individual metabolites and unchanged cyclophosphamide (CPA) in control and phenobarbital-treated animals, using radiolabelled CPA together with thin-layer chromatography. On Day 0, one group was started on phenobarbital drinking water and one group stayed on regular acid water. P388 leukaemia, ($10^6$ cells i.p.) was administered to all mice on Day 8, and 2 days later both groups of mice were given i.p. CPA (200 mg/kg) with $^{14}$C-CPA (0·2 μCi per mouse). At 5–60 min after CPA administration, groups of 10 mice were killed and their blood collected for assay of parent compound and metabolites in plasma. Phenobarbital pretreatment reduced CPA and phosphoramide mustard CXT (concentration × time) by 66+% and 27+%, respectively. Assuming that phosphoramide mustard is both the ultimate cytotoxic form of CPA and the blood-transport form, the reduction of CXT by phenobarbital would predict a decreased therapeutic effect. The assay methods in this study will be used in the future to determine the importance of this potential drug interaction in man.**

THE PATIENT with advanced cancer is treated with a number of supportive medications which may alter hepatic enzyme activity and, consequently, may influence the metabolism and antitumour effects of certain anticancer agents (e.g. cyclophosphamide (CPA) and adriamycin) (Alberts and van Daalen Wetters, 1976; Field *et al.*, 1972; Garratini *et al.*, 1975; Hart and Adamson, 1969; Jao, *et al.*, 1972; Reich and Bachur, 1976; Sladek, 1972). We have been studying the underlying mechanisms and quantitating the effects on tumour and normal tissue toxicity of potentially efficacious or deleterious interactions between CPA and routine medications. Recognition of such deleterious drug interactions may prove important in the design of dosage schedule of CPA and other anticancer agents.

CPA requires enzymatic oxidation *in vivo* to generate alkylating moieties. The drug is activated by hepatic microsomal enzymes, and the active metabolite reaches target sites through systemic circulation. The complex mode of activation and the identification of metabolites of this agent have been the subject of intense study by several laboratories (Alarcon and Meienhofer, 1971; Colvin, *et al.*, 1973; Norpoth, 1969; Sladek, 1973; Voelcker *et al.*, 1976) and the metabolic scheme elucidated by these investigations is summarized in Fig. 1.

Phenobarbital increases hepatic mixed-function-oxidase activity of microsomes and has been shown to decrease total alkylating activity (Friedman and Boger, 1961) of plasma from CPA-treated mice (Alberts and van Daalen Wetters, 1976; Field *et al.*, 1972; Garratini *et al.*, 1975). Simultaneous decreases were observed in

* To whom requests for reprints should be addressed.

FIG. 1.—Metabolism of cyclophosphamide. (CPA)

antitumour effect in some studies (Alberts and van Daalen Wetters, 1976; Field *et al.*, 1972; Garratini *et al.*, 1975) but not in others (Hart and Adamson, 1969; Sladek, 1972). Because of the correlation we observed earlier (Alberts and van Daalen Wetters, 1976) between phenobarbital enzyme induction and decreased cell killing against P388 leukaemia in DBA/2 mice, we have now studied the quantitative pharmacokinetic differences in plasma levels of individual metabolites and unchanged drug in control and phenobarbital-treated animals, using radiolabelled CPA, in an attempt to correlate levels of parent drug and cytotoxic metabolites with the reduction in antitumour effect we observed earlier. This method is superior to observation of total plasma alkylating activity by the Friedman–Boger method (Friedman and Boger, 1961) because the latter method , as normally applied, does not clearly differentiate between agents that alkylate at physiological temperature and pH and those that do not, and, even more importantly, does not assess relative contributions of activated, potentially-active, and deactivated species.

### MATERIALS AND METHODS

*Radioactive cyclophosphamide.*—$^{14}C$-side-chain-labelled CPA was kindly supplied by Dr. Robert R. Engle, Head of Chemical Resources Section, National Cancer Institute, Silver Spring, Md, and was purified by thin-layer chromatography. Sp. act. was 24 $\mu$Ci/mg after purification.

*Mice.*—Six- to 8-week-old male DBA/2 mice (The Jackson Laboratory, Bar Harbor, Maine) weighing ~ 20 g were used in these experiments .

*Chemotherapeutic agents.*—Sodium phenobarbital (U.S.P. Crystalline, Mallinckrodt, St. Louis, Mo.) in parenteral form was brought to a final concentration of 0·5 mg/ml of solution by the addition of acid water. CPA (Mead Johnson, Evansville, Indiana) was dissolved in sterile water in the desired concentration and $^{14}C$-CPA (0·2 $\mu$Ci per mouse) was added.

*The administration schedule of chemotherapeutic agents.*—Mice were divided into 2 groups of 10 animals. On Day 0, one group was started on phenobarbital drinking water and one group stayed on acid water (McPherson, 1963). P388 leukaemia, ($10^6$ cells i.p.) was administered to all mice on Day 8. On Day 10 both groups of mice received i.p. CPA (4 mg per mouse or 200 mg/kg body wt) together with $^{14}C$-CPA (0·2 $\mu$Ci per mouse). Mice averaged 2·5 ml/day of either acid water or phenobarbital drinking water, so that, daily total phenobarbital dosage averaged 1·25 mg or ~ 62·5 mg/kg.

To prove microsomal enzyme induction, control and phenobarbital-pretreated mice underwent sleep-duration studies after pentobarbital administration. Pentobarbital, at a dose of 45 mg/kg, caused acid-water-fed mice to sleep between 25 and 45 min. Those animals pretreated with phenobarbital either did not sleep or rarely slept beyond 5 to 8 min following pentobarbital injection.

*Collection of plasma.*—At each sampling time after CPA administration (5, 10, 15, 20, 45, 60 min) groups of 10 mice were killed by decapitation and their blood collected in iced, heparinized centrifuge tubes. The blood samples were centrifuged at 2000 rev/min for 10 min at 4°C, and the resulting 2·5 ml of plasma (for each group of 10 mice) was immediately separated and frozen at −20°C.

*Preparation of metabolite fractions.*—Plasma fractions were allowed to thaw and immediately extracted with chloroform (3 × 10 ml) followed by methanol (10 ml), leaving a small solid residue that was removed by filtration. The residue was extracted with methanol (2 × 10 ml) by trituration and filtration, and the filtrates were combined with the first methanol extract. Chloroform extracts were evaporated to dryness at water aspirator pressure and stored at −20°C. Methanol extracts were treated with excess diazomethane, allowed to stand 10 min at room temperature, evaporated in a stream of $N_2$, and stored at −20°C.

*Thin-layer chromatography.*—Analysis of plasma fractions was performed in duplicate (half of the total extract being applied to each of 2 plates) on Analtech (Newark, Delaware) precoated silica-gel G plates (250 $\mu$m thickness) in acetone: chloroform (3:1, v/v) for non-polar metabolites and in chloroform: methanol (9:1, v/v) for polar metabolites. Plates were activated by heating 1 h at 100°C and storing in a dessicated cabinet.

*Isolation of metabolites.*—Thin-layer chromatograms were sprayed with a 1% solution of 4-(*p*-nitrobenzyl)pyridine (NBP) in acetone, heated in an oven for 15 min at 140°C, and sprayed with a 3% solution of KOH in methanol. Alkylating components yielded blue spots. Our previous studies (Struck *et al.*, 1975) had identified the alkylating components, and use of synthetic standards permitted identification and collection of individual metabolites.

*Radioactivity determination.—Thin-layer* chromatographic spots were collected from the plates and analysed for radioactivity in AQUASOL (New England Nuclear, Boston, Mass.) after solubilization in methanol with a Packard TRI-CARB Model 3315.

*Pharmacokinetic analysis.* The trapezoidal rule was used to measure the areas under the parent-compound and metabolic-plasma-decay curves.

### RESULTS

A cyclophosphamide (CPA) dose of 200 mg/kg was selected for these studies because it could be sensitively and reproducibly assayed and because we and others had previously used it in mouse studies with this drug (Alberts and van Daalen Wetters, 1976; Field *et al.*, 1972).

Extraction of plasma from mice treated with CPA with chloroform followed by methanol consistently removed ~ 95% of the radioactivity, as illustrated for a single experiment (30 min after drug treatment) in Table I, leaving a non-alkylating residue and indicating that the unextracted radioactivity did not consist of alkylating metabolites. Similar results

TABLE I.—*Relative radioactivity of extracts of plasma from cyclophosphamide (CPA)-treated mice**

| Sample | Control Radio-activity (%) | Phenobarbital Pretreatment Radio-activity (%) |
|---|---|---|
| Chloroform Extract | 52·7 | 45·9 |
| Methanol Extract | 42·1 | 48·7 |
| Residue | 5·2 | 5·4 |

*Blood was collected 30 min after drug treatment.

FIG. 2.—Total plasma levels of CPA and its alkylating metabolites in mice. ——— No phenobarbital pretreatment; – – – – – Phenobarbital pretreatment.

were obtained earlier with whole blood (Struck *et al.*, 1975). At each sampling time, recovery of radioactivity in the 3 fractions amounted to 0·5–1% of the administered dose. Contrasting with these radioactivity results, in every experiment relative alkylating activity of chloroform (non-polar) extracts was much greater than that of methanol (polar) extracts, as judged by colour formation of equivalent amounts of the 2 extracts reaction with NBP (Struck *et al.*, 1975). This observation emphasizes the relatively large amount of non-alkylating, polar metabolites in methanol extracts, although radioactivity differences were small.

The effect of phenobarbital on total plasma levels of unchanged CPA and its alkylating metabolites is illustrated in Fig. 2. Phenobarbital pretreatment produced consistently lower levels of parent

TABLE II.—*Comparative pharmacokinetics of cyclophosphamide (CPA) and its alkylating metabolites before and after phenobarbital microsomal enzyme induction*

| | Area under plasma decay curves (μg min/ml) | | |
|---|---|---|---|
| | Acid-Water Control (1) | Phenobarbital water 62·5 mg/kg* (2) | Ratio 2:1 |
| Total CPA and Metabs. | 821 | 527 | 0·64 |
| CPA | 402 | 131 | 0·33 |
| 4–Keto CPA | 267 | 294 | 1·10 |
| PM† | 36 | 26 | 0·72 |
| CP‡ | 7 | 5 | 0·71 |

*Daily for 10 days

†Phosphoramide mustard

‡Carboxyphosphamide

FIG. 3.—Plasma levels of CPA and its major, non-polar metabolites in mice. ——— No phenobarbital pretreatment; - - - - - - Phenobarbital pretreatment; CPA (○); 4-KetoCPA (□); Nor-HN 2(△).

FIG. 4.—Plasma levels of minor, non-polar metabolites of CPA in mice. ——— No phenobarbital pretreatment; - - - - - - Phenobarbital pretreatment; Dechloroethylcyclophosphamide (○); Alcophosphamide (□).

compound and metabolites in plasma at every sampling point after drug administration, the area under the plasma decay curve being only 58·8% of that found for control mice (Table II). However, differences in levels of the therapeutically efficacious, alkylating components in the non-polar fraction is emphasized when total, major, non-polar metabolites are fractionated. Results are shown in Fig. 3. CPA CXT for the phenobarbital group were only 33·2% of those for control mice, whereas levels of 4-ketoCPA, a major non-polar, inactive, metabolite in blood (Struck *et al.*, 1975), were similar for both groups (Table II).

Fractionation of two minor, non-polar metabolites gave the results shown in Fig. 4. More rapid clearance of these metabolites in the phenobarbital group is apparent.

FIG. 5.—Plasma levels of the major, polar metabolites of CPA in mice. ——— No Phenobarbital pretreatment; - - - - - Phenobarbital pretreatment; Phosphoramide mustard (○); Carboxyphos-phamide (□).

Use of a different thin-layer solvent system has resulted in separation of the 2, major, polar plasma metabolites, phosphoramide mustard and carboxyphosphamide. The methyl esters of these metabolites were separable in chloroform: methanol (9:1), giving $R_f \sim 0{\cdot}55$ for carboxyphosphamide methyl ester and $R_f \sim 0{\cdot}45$ for phosphoramide mustard methyl ester. Plasma levels are shown in Fig. 5. Consistently higher levels of phosphoramide mustard were observed relative to carboxyphosphamide. However, peak levels of phosphoramide mustard are much lower than those of CPA and 4-ketoCPA. Again, CXT areas for the control mice were higher than those for phenobarbital mice (Table II).

### DISCUSSION

By use of a previously described thin-layer chromatographic method (Struck *et al.*, 1975), CPA and its alkylating metabolites in chloroform and methanol extracts of plasma from control and phenobarbital-treated mice were fractionated and identified by co-chromatography with synthetic standards. The extraction procedure recovered 95% of the radioactivity in plasma after administration of $^{14}C$-side-chain-labelled CPA, as well as complete removal of alkylating components, as demonstrated by reaction of the extract residues with 4-*p*-(nitrobenzyl pyridine. This high recovery of radioactivity demonstrates that little covalent binding of CPA or its metabolites containing the labelled moiety occurs with plasma components, and assures that all major metabolites are available in the 2 extracts for identification and quantitation. Identical results were obtained in previous studies (Struck *et al.*, 1975) with both ring- and side-chain-labelled drug.

Thin-layer chromatograms in this study were qualitatively identical to those observed in our earlier studies on pooled blood samples (Struck *et al.*, 1975) and on blood samples from 40 individual mice (Struck, *et al.*, 1977). The earlier, extensive investigation of individual metabolite fractions by mass spectrometric analysis had confirmed the identity of the metabolites, and had revealed that other chlorine-containing, similarly volatile metabolites were not present in these fractions, at least in sufficient concentration to reveal chlorine-isotope peaks that were distinguishable from other peaks in the mass spectra (Struck *et al.*, 1975). Furthermore, since the spectrum of metabolites observed in this study was identical to that in our earlier studies (Struck *et al.*, 1975; Struck *et al.*, 1977) and in studies by others by different methods (Bakke *et al.*, 1972; Colvin *et al.*, 1976; Connors *et al.*, 1974; Voelcker *et al.*, 1976), the likelihood of significant contamination by other, yet unidentified metabolites is low. It is emphasized that, although the electron-impact mass spectral method used in our earlier studies would not confirm the presence of 4-hydroxyCPA, a synthetic specimen was used as a chromatographic standard in this and the earlier studies (Struck *et al.*, 1975; Struck *et al.*, 1977 and this metabolite was not detected in this or in any of the earlier studies. The limit of detection of the alkylating metabolites of CPA by our method is 0·1 μg; for the volume of plasma used in this study (2·5 ml) the limit of detection for duplicate determinations would be approximately 0·08 μg/ml. In earlier studies (Struck *et al.*, 1975) which used 12·5 ml of blood or at least 5 ml equivalents of plasma, the limit of detection would be approximately 0·04 μg/ml for duplicate determinations. Of interest is a recent report by Wagner *et al.*, (1977) of levels of stabilized 4-hydroxyCPA (0·14–0·42 μg/ml) higher than the limiting values cited above, in blood of humans 2 h after an i.v. dose of CPA of only 10 mg/kg, whereas our present and previous studies used doses of 100–300 mg/kg. It is possible, however, that degradation of any 4-hydroxyCPA in our samples could have occurred before chromatography, since stabilization was not attempted. Future studies will employ one or both of the reported stabilization techniques (Fenselau *et al.*, 1977; Wagner *et al.*, 1977) to attempt quantitation of this metabolite, whose role in the overall cytotoxic effect of CPA *in vivo* is still unresolved (Cox *et al.*, 1975; Fenselau *et al.*, 1977; Struck *et al.*, 1975; Wagner *et al.*, 1977).

The effect of phenobarbital on CPA metabolism has been investigated by several groups (Alberts and van Daalen Wetters 1976; Field *et al.*, 1972; Garratini *et al.*, 1975; Hart and Adamson, 1969; Jao *et al.*, 1972; Sladek, 1973). However, none of these prior studies included quantitation of individual polar and nonpolar metabolites. In this study we have identified and quantitated 4-ketoCPA, nor-nitrogen mustard, dechloroethyl-CPA, "alcophosphamide" {3-hydroxypropyl *N*,*N* - bis (2 - chloroethyl )phosphorodiamidase}, phosphoramide mustard, and carboxyphosphamide, as well as parent drug, in plasma of phenobarbital-treated and control mice over a range of time (5–60 min).

An interesting difference at the shorter sampling times between this study and our prior study is the consistently lower levels of the total of unchanged drug and alkylating metabolites in this study (Figs. 2, 3, 4) compared to higher total plasma alkylating activity in the prior study (Alberts and van Daalen Wetters, 1976). This difference may be the result of the method used (Friedman and Boger, 1961) for determination of plasma alkylating activity; the method quantitates alkylation at 100°C for 25 min in acid and is clearly not physiological. As a result, the Friedman–Boger method may inaccurately reflect potential alkylating components in plasma at 37°C and pH 7.

In previous studies (Connors *et al.*, 1974; Struck *et al.*, 1975) all the non-polar metabolites and one polar metabolite (carboxyphosphamide) identified and quantitated in this study were shown to be inactive as antitumour agents. Consequently Fig. 2, which reflects total levels of CPA and its alkylating metabolites, is misleading as an indication of levels of components potentially able to produce an antitumour effect, since it includes metabolites known to be ineffective. Of all the alkylating, radioactive components isolated and identified in the non-polar fraction, only CPA is potentially capable (through subsequent conversion to 4-hydroxyCPA and phosphoramide mustard) of killing tumour cells. Therefore Fig. 3 and Fig. 5, insofar as they show levels of CPA and phosphoramide mustard, are better indicators of the reason for differences in tumour response to CPA, with and without phenobarbital pretreatment.

Peak plasma levels of phosphoramide mustard, probably the ultimate intracellular cytotoxic form of CPA (Colvin *et al.*, 1973; Connors *et al.*, 1974; Struck *et al.*, 1971) [although there is disagreement about the role of this metabolite as a blood transport form in producing the cytotoxic effect of cyclophosphamide (Colvin *et al.*, 1976; Cox *et al.*, 1975; Hohorst *et al.*, 1976; Struck *et al.*, 1975)] and carboxyphosphamide are low (5–10%) in comparison with peak levels of CPA. Data presented in Fig. 5 represent the first quantitation of levels of phosphoramide mustard in mouse plasma after CPA treatment. Voelcker *et al.* (1976) quantitated this metabolite, along with some unidentified metabolites in rat serum, and observed similar levels relative to CPA, and quantitation in human plasma has recently been accomplished (M. Colvin, personal communication). Differences in levels of phosphoramide mustard between the phenobarbital and control groups are small during the first 20 min, after which somewhat higher levels of phosphoramide mustard persist for the remaining 40 min of the study in the control group. It is tempting to speculate that his persistence is the cause of the improved (1 log) cell killing observed in our earlier work (Alberts and van Daalen Wetters, 1976) against the very responsive P388 leukaemia. Indeed, if it is true that the antitumour effect of a cell cycle-nonspecific chemotherapeutic agent correlates with the plasma CXT area of the active form of a drug (Jao *et al.*, 1972; Jusko, 1971; Sladek, 1972) and if phosphoramide mustard is accepted as both the ultimate cytotoxic form of the drug and as the blood-transport form, the areas of control and phenobarbital groups, which indicate a 39% greater area for the control, would predict a decreased therapeutic effect. Whether such a difference is true for humans has not been determined, but should be evaluable by the methods used in this study.

This investigation was supported in part by the Medical Oncology Program Project Grant CA–17094 (to the University of Arizona) and by Contract No 1–CM–43762 (to Southern Research Institute) from the National Cancer Institute, National Institutes of Health, Bethesda, Maryland, and by the General Research Service of the University of Arizona Health Science Center.

### REFERENCES

Alarcon, R. A. & Meienhofer, J. (1971) Formation of cytotoxic aldehyde acrolein during *in vitro* degradation of cyclophosphamide. *Nature, New Biol.*, **233**, 250.

Alberts, D. S. & van Daalen Wetters, T. (1976) The effect of phenobarbital on cyclophosphamide antitumor activity. *Cancer Res.*, **36**, 2785.

Bakke, J. E., Feil, V. J., Fjelstul, C. E. & Thacker, E. J. (1972) Metabolism of cyclophosphamide by sheep. *J. Agr. Food Chem.*, **20**, 384.

Colvin, M., Brundrett, R. B., Kan, M.-N. N., Jardine, I. & Fenselau, C. (1976) Alkylating properties of phosphoramide mustard. *Cancer Res.*, **36**, 1121.

Colvin, M., Padgett, C. A. & Fenselau, C. (1973) A biologically active metabolite of cyclophosphamide. *Cancer Res.*, **33**, 915.

Connors, T. A., Cox, P. A., Farmer, P. B., Foster, A. B. & Jarman, M. (1974) Some studies of the active intermediates formed in the microsomal metabolism of cyclophosphamide and isophosphamide. *Biochem. Pharmacol.*, **23**, 115.

Cox, P. J., Phillips, B. J. & Thomas, P. (1975) The enzymatic basis of the selective action of cyclophosphamide. *Cancer Res.*, **35**, 3755.

Fenselau, C., Kan, M.-N. N., Rao, S. S., Myles, A., Friedman, O. M. & Colvin, M. (1977) Identification of aldophosphamide as a metabolite of cyclophosphamide *in vitro* and *in vivo* in humans. *Cancer Res.*, **37**, 2538.

Field, R. B., Gang, M., Kline, I., Venditti, J. M. & Waravdekar, V. S. (1972) The effect of phenobarbital or 2-diethylaminoethyl 2,2-diphenylvalerate on the activation of cyclophosphamide *in vivo*. *J. Pharmacol. Exp. Therap.*, **180**, 475.

Friedman, O. M. & Boger, E. (1961) Colorimetric estimation of nitrogen mustards in aqueous media. *Anal. Chem.*, **33**, 906.

Garratini, S., Bartosek, I., Donelli, M. G. & Speafico, F. (1975) Interactions of anticancer agents with other drugs. In *Pharmacologic Basis of Cancer Chemotherapy, Baltimore: Williams and Wilkins*. 565.

Hart, L G. & Adamson, R. H. (1969) Effect of microsomal enzyme modifiers on toxicity and therapeutic activity of cyclophosphamide in mice. *Arch. Int. Pharmacodyn. Ther*, **180**, 391.

Hohorst, H.-J., Draeger, U., Peter, G. & Voelcker, G. (1976) The problem of oncostatic specificity of cyclophosphamide (NSC-26271). studies on reactions that control the alkylating and cytotoxic activity. *Cancer Treat. Rep.*, **60**, 309.

Jao, J. Y., Jusko, W. J. & Cohen, J. L. (1972) Phenobarbital effects on cyclophosphamide pharmacokinetics in man. *Cancer Res.*, **32**, 2761.

Jusko, W. J. (1971) Pharmacodynamics of chemotherapeutic effects: dose–time–response relationships for phase-nonspecific agents. *J. Pharm. Sci.*, **60**, 892.

McPherson, C. W. (1963) Reduction of *Pseudomonas aeruginosa* and coliform bacteria in mouse drinking water following treatment with hydrochloric acid or chlorine. *Lab. Animal Care*, **13**, 737.

Norpoth, K. (1969) Untersuchungen zur oxidativen Umsetzung von Endoxan *in vivo* und *in vitro*. Münster, Habilitationsschrift, Fach Physiologische Chemie, Wesfalischen Wilhelms-Universität.

Reich, S. D. & Bachur, N. R. (1976) Alterations in Adriamycin efficacy by phenobarbital. *Cancer Res.*, **36**, 3803.

Sladek, N. E. (1972) Therapeutic efficacy of cyclophosphamide as a function of its metabolism. *Cancer Res.*, **32**, 535.

SLADEK, N. E. (1973) Evidence for an aldyhyde possessing alkylating activity as the primary metabolite of cyclophosphamide. *Cancer Res.*, **33**, 651.

STRUCK, R. F., KIRK, M. C., MELLETT, L. B., EL DAREER, S. & HILL, D. L. (1971) Urinary metabolites of the antitumors agent cyclophosphamide. *Mol. Pharmacol.*, **7**, 519.

STRUCK, R. F., KIRK, M. C., WITT, M. H. & LASTER, W. R., Jr. (1975) Isolation and mass spectral identification of blood metabolites of cyclophosphamide: evidence for phosphoramide mustard as the biologically active metabolite. *Biomed. Mass Spectr.*, **2**, 46.

STRUCK, R. F., ROSE, W. C. & SCHABEL, F. M., Jr. (1977) Attempts to develop a predictive method for response of ridgway osteogenic sarcoma (ROS) to cyclophosphamide (CPA) therapy. *Proc. Am. Assoc. Cancer Res.*, **18**, 45.

VOELCKER, G., WAGNER, T. & HOHORST, H.-J. (1976) Identification and pharmacokinetics of cyclophosphamide (NSC-26271) metabolites *in vivo*. *Cancer Treat. Rep.*, **60**, 415.

WAGNER, T., PETER, G., VOELCKER, G. & HOHORST, H.-J. (1977) Characterization and quantitative estimation of activated cyclophosphamide in blood and urine. *Cancer Res.*, **37**, 2592.